# Prospective prediction of first onset of major depressive disorder in midlife using machine learning

**DOI:** 10.1007/s00127-025-02942-z

**Published:** 2025-06-18

**Authors:** Johannes Massell, Martin Preisig, Marcel Miché, Marie-Pierre F. Strippoli, Giorgio Pistis, Roselind Lieb

**Affiliations:** 1 Division of Clinical Psychology and Epidemiology, Department of Psychology, University of Basel, Missionsstrasse 62a, Basel, 4055 Switzerland; 2 Psychiatric Epidemiology and Psychopathology Research Center, Department of Psychiatry, Lausanne University Hospital and University of Lausanne, Rte de Cery 25, 1008 Prilly, Switzerland

**Keywords:** Major depressive disorder, Prospective prediction, Machine learning, Clinical utility

## Abstract

**Purpose:**

In this paper we leverage machine learning (ML) models to prospectively predict the first onset of Major Depressive Disorder (MDD), one of the most common and disabling mental health conditions. While such prediction models hold potential for enabling early interventions, few studies have applied ML approaches to this task, and those that have are heterogeneous in nature. Moreover, the clinical utility of these predictive models remains largely unexamined.

**Methods:**

Data stemmed from CoLaus|PsyCoLaus, a population-based cohort study. In total, 1350 participants, age 35–66 years without lifetime MDD at baseline participated in the physical and psychiatric baseline and at least one psychiatric follow-up evaluation. Models based on logistic regression, elastic net, random forests, and XGBoost were trained using an extensive array of psychosocial, environmental, biological, and genetic predictors. Discriminative performance, calibration, clinical utility, and individual predictor contributions were assessed using nested cross-validation.

**Results:**

Discriminative performance was comparable between models (areas under the precision-recall curve between 0.36 and 0.38; areas under the receiver operating characteristic curve between 0.65 and 0.68). Decision curve analysis suggested clinical utility of logistic regression, elastic net, and random forests for threshold probabilities between 10% and 40%. Across all models, neuroticism, sex, and age were the most important predictors.

**Conclusions:**

Although the prediction models achieved discriminative performance levels above chance, further refinement is necessary. The addition of biological and genetic predictors did not elevate performance markedly. Additional research seems warranted given the limited number and heterogeneous nature of existing studies, the burden associated with MDD, and the potential to improve overall outcomes for people at risk for MDD.

**Supplementary Information:**

The online version contains supplementary material available at 10.1007/s00127-025-02942-z.

## Introduction

### Major depressive disorder

Annually, an estimated 38.2% of the European Union population suffers from one or more mental disorders, which poses a significant burden on affected individuals, their immediate environment, and society. Major depressive disorder (MDD) is not only among the most prevalent but also one of the most debilitating mental disorders [1]. MDD is characterized by persistently depressed mood and diminished interest in typically enjoyable activities. Additionally, it often involves changes in body weight, cognitive impairments, fatigue, low self-esteem, and disrupted sleep patterns [2]. There is consensus regarding the multifactorial etiology of MDD [3]. For example, the biopsychosocial model of depression posits that biological (e.g., genetic vulnerability), psychological (e.g., maladaptive coping strategies), and social (e.g., childhood trauma) factors all play significant roles in the context of MDD [4]. Unfortunately, the rates of response to evidence-based treatments are far from ideal. Estimates indicate that between one-third and two-thirds of patients treated for MDD do not adequately respond to an initial psychopharmacological treatment attempt, and between 15% and 33% of patients continue to exhibit inadequate responses even after multiple treatment attempts [5].

Many risk factors for MDD with varying effect sizes (e.g., physical abuse during childhood, obesity, sexual dysfunction) have been suggested in the literature [6], and some of this research also directly precedes and informs the present study [7–10]. A previous prospective analysis of the CoLaus|PsyCoLaus study based on multinomial logistic regression and a follow-up of more than 5 years identified a series of predictors for the subtypes of MDD [9]. These significant predictors included female sex, depressive syndromes below the threshold of MDD and neuroticism scores for both the atypical and the melancholic subtypes, BMI levels for the atypical subtype only and absence of hypertension for the melancholic subtype. Similar results have been documented in the NESARC study, a large prospective population-based cohort study conducted in the US [11].

Far less is known, however, about whether these established risk factors can be leveraged to prospectively predict the onset of MDD. The core of the present study is therefore prediction modeling rather than explanatory modeling [12]. Timely identification of at-risk individuals with such prediction models could facilitate early interventions and has the potential to improve overall outcomes for people at risk for depression [13–15].

### Machine learning

Machine learning (ML) encompasses various algorithms (e.g., random forests, support vector machines, and artificial neural networks) designed to identify patterns in data and subsequently make individual predictions for previously unseen cases. This shift in focus from group-level inference to individual-level predictions serves as the foundation for the emerging fields of precision medicine and psychiatry. Further, contemporary ML frameworks harness the power of today’s advanced computing capabilities and offer sophisticated workflows for internal validation, effectively preventing overoptimistic prediction models that perform well only within the sample they were trained on and not outside of it [16]. The use of ML in the fields of clinical psychology and psychiatry is still in its infancy with many ML studies still suffering from suboptimal methodology [17]. Nonetheless, in a world with ever-increasing amounts of complex data, ML has established itself as an indispensable tool and has already demonstrated promising potential in the diagnosis, prognosis, and treatment of mental disorders [18].

### Prospective prediction of first onset of MDD using ML

Although there are numerous published studies investigating risk factors for depression [6], only a few rather heterogeneous studies have used ML to prospectively predict the first onset of depression or MDD (see Tables S1 and S2 in the supplementary materials). The discriminative performance of the reported prediction models varies greatly and ranges from “bad” to “excellent” [19], as indicated by area under the receiver operating characteristic curve (AUC) values between 0.55 and 0.94. Most of the existing studies investigated very specific populations such as (child) soldiers [20, 21], Parkinson’s patients [22], or civil servants [23], with only one study incorporating a population sample from a representative household survey [24]. The age ranges of the participants are often very restrictive, usually focusing on either early [20, 25] or late [26–28] stages of life, with a mere four studies that included a broader age range in adulthood [21–24]. Almost all studies relied exclusively on sociodemographic, psychosocial, and clinical predictors stemming from either self-report questionnaires or interviews. Only two studies additionally incorporated biological predictors stemming from blood and urine samples [26] and saliva samples [25]. Common methodological limitations such as small sample sizes, potentially overoptimistic validation procedures, and sparse reporting of performance metrics further constrain the current understanding of prospective prediction of first onset of depression/MDD. Only one of these studies [28] addressed whether their prediction models would be suitable for clinical practice by evaluating clinical utility. Last, all investigated samples are from the Americas or Asia, underscoring the need for an investigation involving a European cohort.

### Aims of the current study

Using data from a population-based cohort study relying on semi-structured diagnostic interviews as well as thorough physical and biochemical investigations, our goal was to (1) expand the limited understanding of first onset of MDD prediction, by evaluating and comparing multiple ML-based prediction models, trained using an extensive array of candidate predictors including sociodemographic, behavioral, psychological and inflammatory variables, traumatic events, pre-existing psychiatric and cardio-metabolic disorders as well as family history for MDD and polygenic risk score for MDD; (2) explore the contributions of individual predictors to the predictive performances of our models and (3) assess the clinical utility of our prediction models in terms of net benefit (NB) via decision curve analysis (DCA). In contrast to previous analyses [9], the present study focused on prediction modeling of MDD rather than explanatory modeling of MDD subtypes. Moreover, the present analyses included (1) continuous rather than categorical variables for systolic blood pressure [SBP], high-density lipoprotein [HDL] cholesterol and triglycerides; and (2) additional variables for marital status, extraversion, psychoticism, and a polygenic risk score (PRS) for MDD.

## Materials and methods

### Study design and sample

**Fig. 1:**
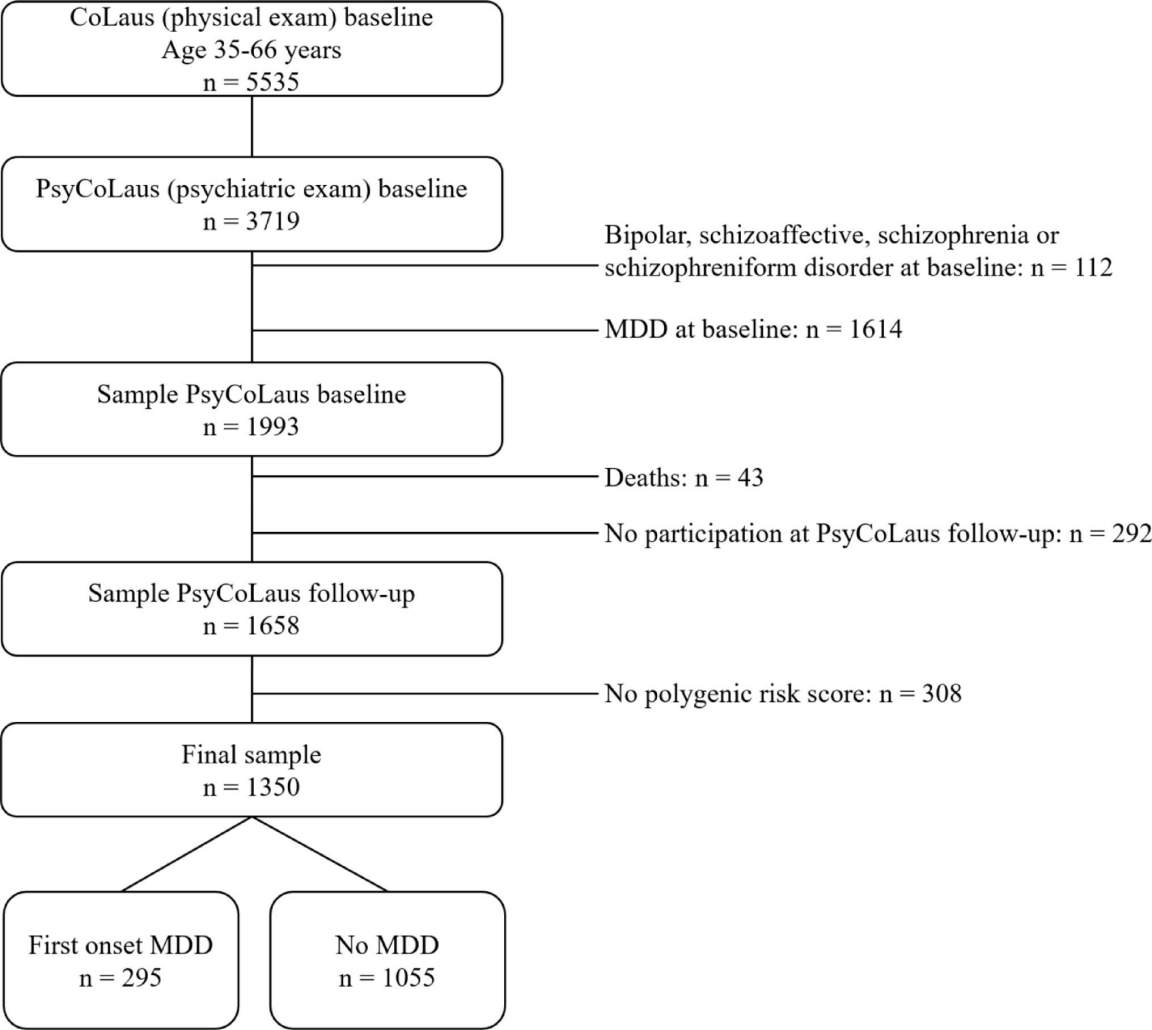
Flow chart of included participants.* MDD* Major depressive disorder

Data stem from the CoLaus|PsyCoLaus study [7, 8], a prospective cohort investigating mental disorders and cardiovascular risk factors in a community sample. Participants were randomly selected from 35- to 75-year-old residents of Lausanne, Switzerland, between 2003 and 2006. Following baseline physical and psychiatric assessments (2003–2008), three follow-ups were conducted at approximately 5, 10, and 14 years. Of the 5,535 participants completing the physical exam, 67% (aged 35–66) also underwent psychiatric evaluation (Fig. 1). Individuals with lifetime diagnoses of MDD, bipolar disorder, or psychotic disorders at baseline were excluded. Among the remaining 1,993 subjects, 43 died during follow-up, and 1,658 (83% of survivors) participated in at least one psychiatric follow-up. Due to genetic data availability only for Caucasians, 308 participants were excluded, resulting in a final sample of *n* = 1,350. Nonparticipants at follow-up had lower socioeconomic status and were less likely to report a family history of MDD.

### Measures

The instruments used for the assessment of the outcome variable (MDD) and candidate predictors at baseline and their psychometric properties are described in detail in the supplementary materials (SM1: Measures).

This study is based on secondary data, meaning that the variables in our models were originally collected within the framework of the broader CoLaus|PsyCoLaus study, rather than being gathered specifically for the current research aims. Of the hundreds of variables assessed for CoLaus|PsyCoLaus, we identified and selected 29 candidate predictors a priori. All included candidate predictors have been associated with MDD in the literature. The final predictor pool used for the present analyses consisted of 12 categorical candidate predictors at baseline—sex, marital status, relatives with MDD, dysthymia, other specified depressive disorders (brief depression, recurrent brief depression, and minor depression), alcohol use (abuse or dependence) disorders, drug use (abuse or dependence) disorders, anxiety disorders (panic disorder, agoraphobia, generalized anxiety disorder, social phobia), childhood traumatic events, diabetes, physical inactivity, and current smoking—and 17 continuous candidate predictors at baseline—age, SES, body mass index (BMI), HDL cholesterol, triglycerides, SBP, logarithmic interleukin-6 (IL-6), logarithmic interleukin-1β (IL-1β), logarithmic tumor necrosis factor-α (TNF-α), logarithmic high-sensitivity C-reactive protein (hs-CRP), logarithmic adult life-event negative impact score, neuroticism, extraversion, psychoticism, problem-solving coping, help seeking, and PRS for depression (based on a genome-wide association study (GWAS) *p*-value threshold [pt] of ≤ 0.05).

### Statistical analysis

All analyses were conducted with the ML framework *mlr3* 0.14.1 [29], in R 4.2.2 [30], running within RStudio 2022.12.0.353 [31].

### Prediction models

We developed and validated four prediction models, trained on all predictors simultaneously, to predict first onset of MDD: logistic regression, elastic net [32], random forest [33], and XGBoost [34]. Logistic regression served as a linear baseline, elastic net added regularization and variable selection, while random forest and XGBoost were used to capture non-linear patterns and interactions. By comparing performance and consistency across these complementary models, we aimed to reduce the risk of model-specific bias and increase confidence in the generalizability of our results.

### Performance measures

Model performance was assessed using standard metrics for discrimination and calibration. Discriminative ability was evaluated using the area under the precision-recall curve (PRAUC), area under the receiver operating characteristic curve (AUC), accuracy (ACC), true positive rate (TPR; sensitivity/recall), true negative rate (TNR; specificity), positive predictive value (PPV; precision), negative predictive value (NPV), and the Brier score. Calibration was assessed using the Brier score and calibration plots generated with the probably package [35]. All performance metrics were calculated using a default probability threshold of 0.5, except for AUC and PRAUC, which reflect performance across all possible thresholds.

### Cross-validation and hyperparameter tuning

We implemented repeated nested stratified cross-validation to avoid bias in performance estimation [36, 37]. The inner loop (4-fold, 25 repetitions) was used for hyperparameter tuning via random search, following Bischl et al. [38], with PRAUC as the optimization metric. Logistic regression did not require tuning. The outer loop (10-fold, 10 repetitions) was used for performance estimation. Preprocessing steps, including imputation of missing values [39] and scaling, were applied in each iteration of the outer loop using mlr3pipelines [40].

### Predictor importance

We assessed predictor importance using model-agnostic permutation methods from the iml package [41], based on models trained on MICE-imputed data [42] and validated with repeated stratified 10-fold cross-validation.

### Clinical utility

We assessed clinical utility using Decision Curve Analysis (DCA) with the dcurves package [43]. DCA estimates net benefit (NB) across threshold probabilities (TRPs), comparing each model to default strategies of “treat all” and “treat none” [44, 45]. Following Kessler et al. [46], we considered a TRP range of 10–40%, reflecting half to twice the 22% incidence rate of first-onset MDD in our sample. Such a range is robust against miscalibration and is recommended in the absence of established clinical thresholds [47, 48].

### Robustness check

To verify the impact of imputation, all analyses were repeated using a complete-case dataset after listwise deletion.

## Results

### Descriptive statistics

A total of 295 participants (21.85%) experienced a first onset of MDD during the follow-up period. Characteristics of participants at baseline are reported in Table 1.

**Table 1:** Characteristics of participants at baseline

Variable	Mean/Median/*n*	SD/IQR/%	Missing
Sociodemographic characteristic			
Female, n (%)	593	(43.9)	0
Age (years), mean (SD)	51.6	(8.9)	0
Socioeconomic status^a^, mean (SD)	3.4	(1.3)	0
Married, n (%)	906	(67.1)	0
Family history			
Relatives with MDD, n (%)	419	(33.8)	111
Other lifetime diagnosis			
Dysthymia, n (%)	46	(3.4)	0
OSDD^b^, n (%)	241	(17.9)	0
Alcohol use disorders, n (%)	153	(11.4)	2
Drug use disorders, n (%)	64	(4.7)	0
Anxiety disorders^c^, n (%)	155	(11.5)	0
Cardio-metabolic risk factor			
BMI (kg/m^2^), mean (SD)	25.4	(4.2)	0
Diabetes, n (%)	71	(5.3)	1
HDL cholesterol (mmol/L)^d^, mean (SD)	1.6	(0.4)	1
Triglycerides (mmol/L)^d^, mean (SD)	1.4	(1)	1
SBP (mmHg)^d^, mean (SD)	128	(18)	0
Interleukin-1β, median (IQR)	−0.9	(2.9)	16
Interleukin-6, median (IQR)	0.3	(1.7)	16
TNF-α, median (IQR)	1	(0.9)	16
hs-CRP, median (IQR)	0.1	(1.5)	45
Lifetime event			
Childhood traumatic events^e^, n (%)	66	(4.9)	0
Adult life-event negative impact score, median (IQR)	5.5	(1.2)	10
Behavioral factor			
Current smoking, n (%)	341	(25.3)	0
Physical inactivity^f^, n (%)	542	(40.2)	0
Personality			
Neuroticism, mean (SD)	7.7	(5)	307
Extraversion, mean (SD)	12.3	(4.8)	307
Psychoticism, mean (SD)	5.7	(3.1)	307
Coping			
Problem-solving coping, mean (SD)	7.7	(1.8)	319
Help-seeking, mean (SD)	4.1	(2.6)	319
Polygenic risk score			
Polygenic risk score for depression (p-value cut-off 0.05), mean (SD)	−79.1	(1.3)	0

Descriptive statistics are presented as mean (SD) for continuous variables, median (IQR) for log-transformed continuous variables, and frequency (percentage) for categorical variables.* IQR* Interquartile range;* MDD* Major depressive disorder;* BMI* Body mass index;* SBP* Systolic blood pressure;* HDL* high-density lipoprotein;* TFN- α * tumor necrosis factor-α; hs-CRP = high-sensitivity C-reactive protein;* OSDD* other specified depressive disorders

^a^A value of 3 represents a socioeconomic status of III (middle class) on the Hollingshead scale; ^b^Brief depression, recurrent brief depression, and minor depression; ^c^Panic disorder, agoraphobia, generalized anxiety disorder, and social phobia; ^d^Corrected for medication; ^e^Accident, crime, witness, war, sexual abuse, physical abuse; ^f^Less than 20 min twice a week

### Model performance

**Fig. 2:**
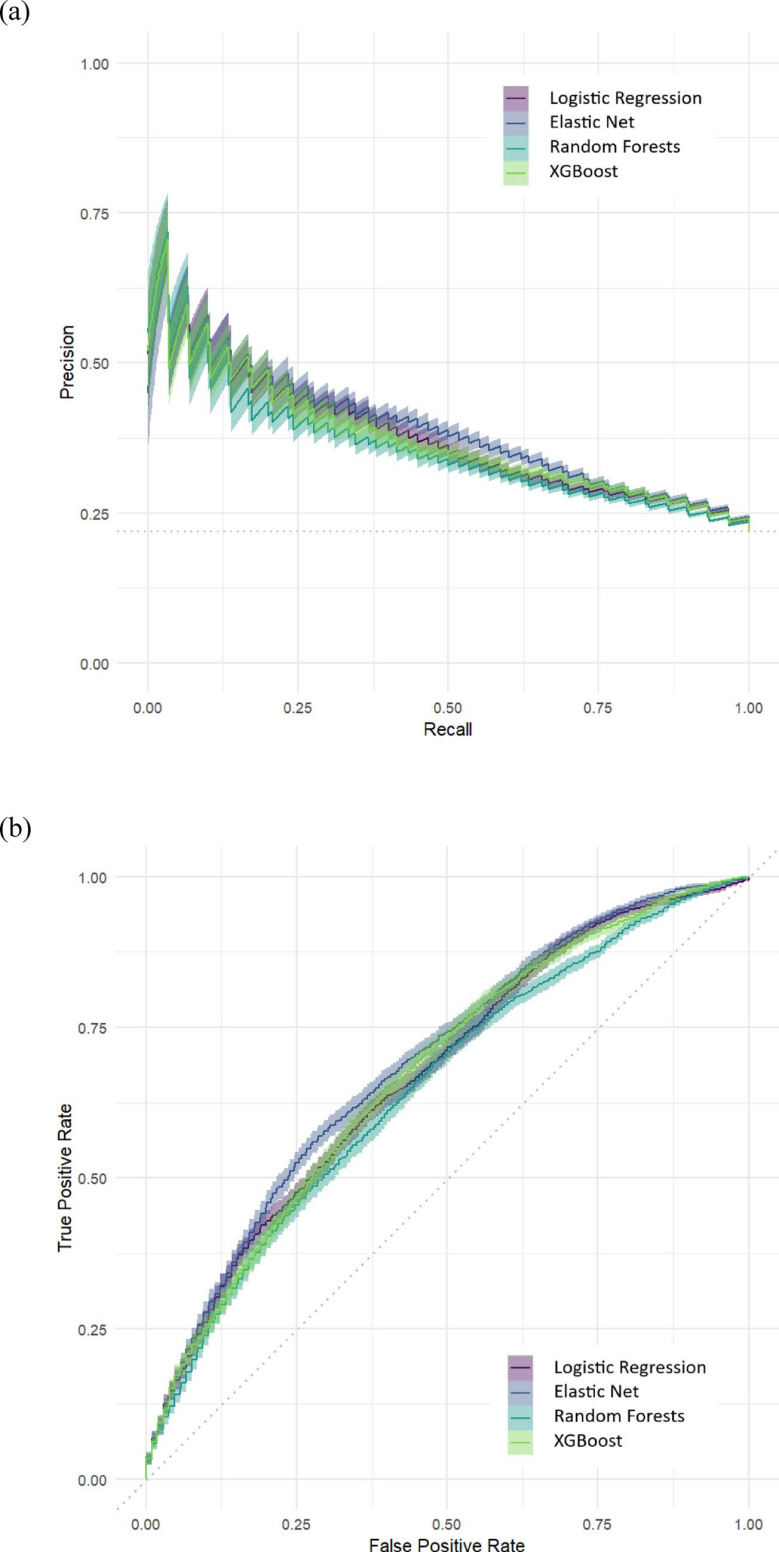
Aggregated model performance metrics based on nested cross-validation procedure. (**a**) Areas under the precision-recall curve (**b**) Areas under the receiver operating characteristic curve.* Note*: Solid lines represent micro averaged values, error bands represent ranges, and dotted lines represent chance classifiers

The means of PRAUC, AUC, ACC, TPR, TNR, PPV, NPV, and BRIER, aggregated over 100 outer resampling iterations for each prediction model, are reported in Table 2. In terms of PRAUC and AUC, the elastic net model demonstrated slightly superior performance, followed by a tie between logistic regression and XGBoost, with random forests slightly lagging behind. The BRIER was identical among logistic regression, elastic net, and random forests, whereas XGBoost showed a slightly inferior score. Overall, the aggregated performance across the four models was remarkably similar for all reported measures. This similarity becomes especially evident when examining the variance over the 100 reported iterations, as illustrated in the boxplots for PRAUC and BRIER in Supplementary Figs. S1 and S2 and in the PRAUC and AUC curves depicted in Fig. 2. Depending on the hyperparameter configuration and variation introduced with each split of the dataset in the cross-validation procedure, each model had the potential to marginally outperform the others. The hyperparameters that we tuned, the ranges of the corresponding search spaces, and the aggregated hyperparameter values used in the 100 outer resampling iterations are presented in Supplementary Table S3.

**Table 2:** Aggregated model performance metrics based on nested cross-validation procedure

Model	PRAUC	AUC	ACC	TPR	TNR	PPV	NPV	BRIER
Logistic regression	0.37	0.67	0.78	0.13	0.96	0.49	0.79	0.16
Elastic net	0.38	0.68	0.78	0.03	0.99	0.52^a^	0.79	0.16
Random forests	0.36	0.65	0.78	0.03	0.99	0.49^a^	0.79	0.16
XGBoost	0.37	0.67	0.78	0.02	0.99	0.46^a^	0.78	0.17

*PRAUC * Area under the precision-recall curve;* AUC* Area under the receiver operating characteristic curve;* ACC *Accuracy;* TPR* True positive rate;* TNR* True negative rate;* PPV *Positive predictive value;* NPV* Negative predictive value;* BRIER* Binary Brier score

^a^Mean positive predictive value for the subset of model fits that did not return “not a number” due to not classifying any individual as belonging to the positive group

### Sensitivity and positive predictive value

At the default probability threshold of 0.5, all four models infrequently predict the positive class (first onset of MDD after baseline) correctly, as indicated by mean TPR (sensitivity) values ranging from 0.02 to 0.13 and mean PPV values ranging from 0.46 to 0.52. On the other hand, all four models frequently predict the negative class (no first onset of MDD after baseline) correctly, as indicated by mean TNR (specificity) values ranging from 0.96 to 0.99 and mean NPV values ranging from 0.78 to 0.79 (Table 2).

### Model calibration

**Fig. 3:**
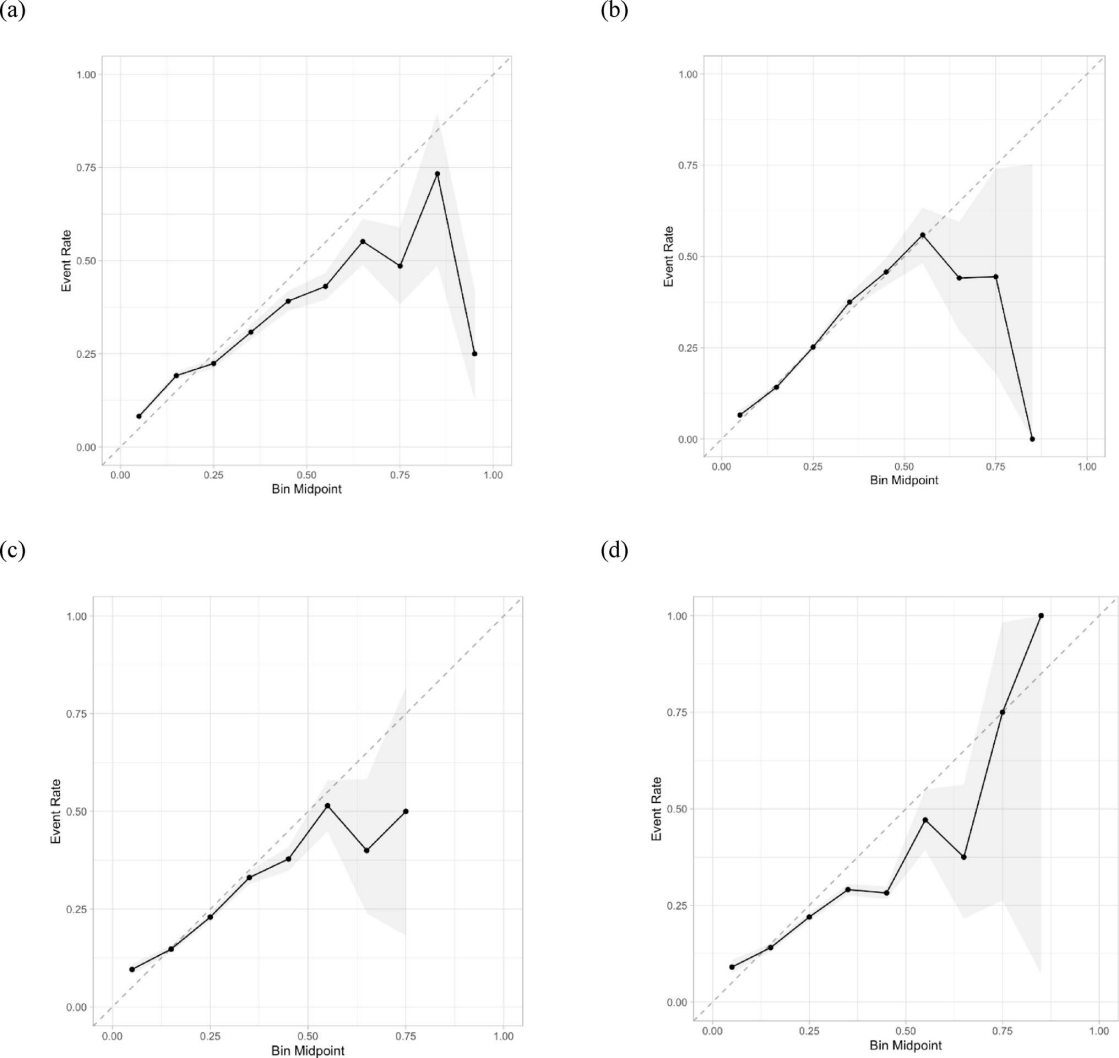
Aggregated model calibration plots based on nested cross-validation procedure. (**a**) Logistic regression (**b**) Elastic net (**c**) Random forests (**d**) XGBoost.* Note*: Points represent tenths of the population divided according to probabilistic predictions (bins). Gray ribbons represent 90% confidence intervals. Dotted lines represent perfect calibration

Model calibration plots for each of the four prediction models are presented in Fig. 3. A perfectly calibrated model is fully congruent with the dotted diagonal. All models demonstrate deteriorating calibration with increasing predicted probabilities. Furthermore, at predicted probabilities of 0.5 and higher, models tend to overpredict risks, except for the XGBoost model, which underpredicts risk within the highest class of predicted probabilities.

### Predictor importance

**Fig. 4:**
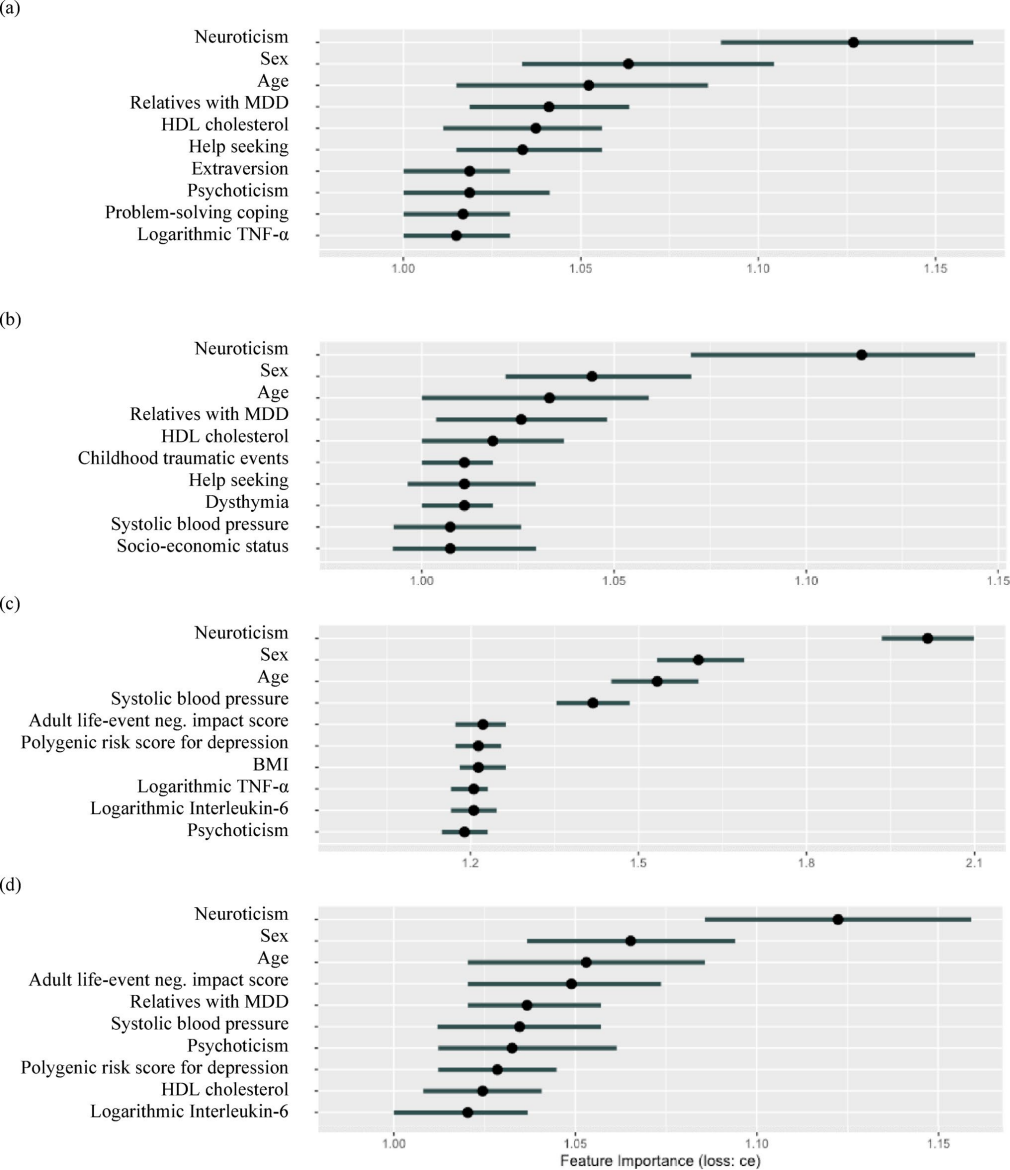
Aggregated predictor importance based on 10-fold cross-validation procedure. (**a**) Logistic regression (**b**) Elastic net (**c**) Random forests. (**d**) XGBoost* Note: MDD* Major depressive disorder;* HDL* high-density lipoprotein;* TNF-α* tumor necrosis factor alpha;* BMI* body mass index;* ce* classification error. Points represent median feature importance and error bars represent 5% and 95% quantiles over 100 permutations of each predictor

For each model, the top 10 predictors with the highest individual contribution are presented in Fig. 4. The permutation of important predictors leads to an increase in classification error, whereas unimportant predictors do not affect the classification error upon permutation. For instance, the permutation of the neuroticism predictor would result in a median increase in the classification error of the logistic regression model by a factor of approximately 1.125. Although each model uses a distinct set of predictors, there is a commonality among all models concerning some of the most important predictors. Neuroticism stands out as the single most important predictor across all models. Additionally, sex and age are key predictors consistently contributing to the performance of all four models.

### Clinical utility

**Fig. 5:**
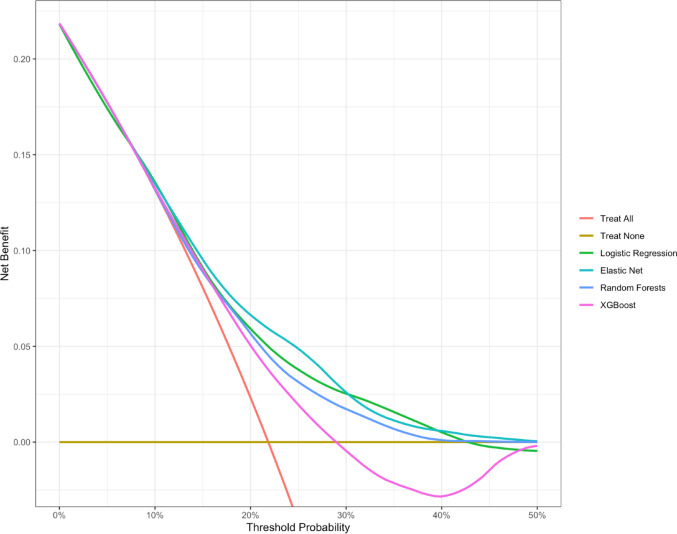
Aggregated decision curve analysis based on nested cross-validation procedure

Figure 5 displays the results of our DCA, facilitating a comparative assessment of the NBs of our four prediction models and the default strategies treat all and treat none. When compared with the treat all strategy (represented by the red line), logistic regression, elastic net, and random forests demonstrate superior NB across the TRP range of 15–40%. In the range between 10% and 15%, all models demonstrate only marginally superior NB. XGBoost shows an increased NB between 15% and 28%, after which it demonstrates clinical harm, compared to the treat none strategy. At the TRP of 22%, which aligns with the incidence rate, the NB range of our models is approximately between 0.03 and 0.07. In practical terms, using our prediction models at this TRP would yield an estimated three to seven additional true positives per 100 predictions without increasing the number of false positives, when compared to the default strategies of treating all and treating none. The NBs of logistic regression, elastic net, and random forests range from about 0.07 (at a TRP of 22%) to 0.01 (at TRPs of 10% and 40%). This implies that the application of these three prediction models in this TRP range is estimated to yield one to seven additional true positives per 100 predictions without incurring an increase in false positives, suggesting clinical utility over the entire TRP range of interest.

### Robustness check

The numerical results when listwise deletion is used instead of imputation, conducted as a robustness check, are reported in SM2 and Tables S4 and S5 in the supplementary materials. An initial review indicates that the results from this robustness check align closely with the findings reported in the main manuscript. This concurrence suggests that the imputation process did not introduce problematic distortion to our results.

## Discussion

Our study was aimed at augmenting existing literature by prospectively predicting first onset of MDD in an adult cohort age 35–66 years old, located in Lausanne, Switzerland. We used prediction models based on logistic regression, elastic net, random forests, and XGBoost, all of which incorporated a comprehensive array of psychosocial, environmental, biological, and genetic predictors. To avoid overconfidence in performance results, we employed a repeated nested stratified cross-validation procedure, promoting a strict separation between model tuning and performance evaluation. The discriminative performances across all four models displayed remarkable similarity, with PRAUC values ranging from 0.36 to 0.38 and AUC values between 0.65 and 0.68. Although the predictive performance of these models certainly surpasses random chance, it remains far from perfect, as AUC values within the 0.6 to 0.7 range signify “sufficient” prognostic accuracy [19]. The probabilistic predictions of our models in part do not match corresponding outcome frequencies found in the real world. Whereas lower predicted probabilities between 0 and 0.4 tend to align with the calibration diagonal, higher predicted probabilities tend to deviate from the calibration diagonal. Additional calibration, especially of models that do not belong to the family of logistic regression, would likely improve this aspect of model performance [49]. However, this requires more data with which to cross-validate the additional calibration. Across all models, neuroticism emerged as the most important predictor, followed by sex and age, which also contributed to the performance of each of the four models. However, for the remaining predictors, the models differed significantly from one another, preventing us from making overarching statements about their importance across models. We demonstrated the clinical utility of the logistic regression, elastic net, and random forests models but cannot recommend using XGBoost as it can cause clinical harm for a range of TRPs of interest. Using our models to guide clinical decisions on whether to recommend prevention efforts within the TRP range of 10–40% would yield a higher number of true positives compared to universally recommending prevention efforts to all individuals or refraining from recommending them to anyone.

To the best of our knowledge only four studies have applied machine learning to prospectively predict the first onset of MDD in samples that include individuals in midlife [21–24]. Two of these focused on highly specific populations, namely patients with Parkinson’s disease [22] and military personnel [21], limiting their comparability to our general population sample. The remaining two studies, conducted in broader cohorts with age ranges of 18 + and 35–74 years, offer the most sensible opportunities for comparison. These studies reported AUC values of 0.76 [24] and 0.71 [23], which are in line with our findings and suggest that prospective prediction of the first onset of MDD in midlife seems feasible but remains moderate in accuracy. These studies do not appear to have employed nested resampling to separate model tuning from performance evaluation, which may have led to overconfident performance estimates. Furthermore, variations in available predictor sets, sample and study characteristics, and outcome definitions (e.g., in each study a different diagnostic interview was used to assess MDD) are also likely to contribute to the slight differences in reported predictive performances across studies. The prognostic accuracy reported in studies with less comparable designs, age ranges, or populations (as summarized in Supplementary Tables S1 and S2) varies widely, ranging from “poor” to “excellent” [19]. This considerable variability highlights not only the potential of machine learning approaches in predicting the (first) onset of depression and MDD in some studies, but also the significant methodological and conceptual challenges that persist. Differences in sample characteristics, predictor selection, outcome definitions, and validation strategies likely contribute to this heterogeneity, emphasizing the need for standardized approaches and cautious interpretation of results.

In our analyses, biological and genetic predictors such as BMI, various inflammatory markers, or the polygenic risk score for depression did not consistently enhance model performance. Although associations between some of these biological predictors and specific MDD subtypes have been previously established [9], these associations did not lead to a substantial improvement in the predictive performance of our models when considering all individuals who experienced a first onset of MDD during the follow-up period collectively. Nevertheless, this process of distilling numerous promising candidate predictors down to a few key ones contributes to filling existing gaps in the literature. Interestingly, despite having access to an unusually broad range of predictors, the empirical results of the random forests and XGBoost models are only just as good as (discriminative performance) or even worse than (clinical utility) the results of the logistic regression and elastic net models. This suggests that complex interactions between predictors did either not exist or, if they existed, did not contribute to improved predictive performance of these tree-based ensemble models.

To date, only one community-based longitudinal study involving individuals aged 65 and above has evaluated the clinical utility of their depression prediction models in terms of NB [28]. When compared with the DCA results of this study, the prediction models trained on our sample demonstrated marginally higher NBs within the TRP range of 10–40%. The ultimate decision on whether our prediction models should, following external validation, be implemented in clinical practice, rests with clinicians in the field. The appropriate harm–benefit ratio, which informs suitable TRP ranges, depends on various clinical factors. These include the nature of the intervention suggested by the model-supported decision, the perceived effectiveness of the suggested intervention in preventing or mitigating the outcome, the availability of clinical resources to administer the intervention, and patient preference, among others. Our findings highlight the broader challenges of achieving clinical utility with predictive models in mental health research. As discussed by Karvelis et al. [50], predictive models often face a gap between statistical performance and practical applicability in real-world clinical settings. This is partly due to the limited reliability of diagnoses of mental disorders and predictor variables, but also because small effect sizes and complex, heterogeneous disorders like MDD reduce the likelihood that predictions will meaningfully inform individual patient care. Even when models demonstrate prognostic accuracy on paper, their added value over existing clinical assessments may be minimal. These considerations emphasize that beyond improving predictive performance, future research must also address how models can be integrated into clinical workflows, offer actionable insights, and balance complexity with interpretability to truly enhance clinical decision-making.

The most salient limitation of our study pertains to the potential for recall bias [51] acting as a confounder across all lifetime measures assessed, a concern that is particularly relevant given the age range of our participants. This limitation is especially noteworthy given the association between neuroticism, identified as the most influential predictor in all our models, and depression [52]. Although we excluded participants with a lifetime diagnosis of MDD from the analyses, the possibility of participants not accurately remembering or misclassifying earlier episodes of MDD cannot be ruled out. In such cases neuroticism would act as proxy for a forgotten episode of MDD and this consideration underscores the need for cautious interpretation when examining the relationship between neuroticism and depression in our study. Second, some variables, such as psychoticism, have weak reliability, which may further limit predictive performance. We recognize that measurement challenges are inherent in mental health research, and even with validated instruments, such limitations should be considered when interpreting our findings. Third, although the predictive performances of the models surpass chance level, inherent heterogeneity of MDD or unmeasured other variables (e.g., a measure of sleep quality) might have limited the performance of our models. Fourth, the restriction to Caucasians from urban areas in this study limits the generalizability of our findings. Fifth, this study was based on secondary data. While this approach allowed us to leverage a rich, existing dataset to investigate the prediction of MDD onset, it also comes with inherent limitations. The data were not originally collected to address our specific research questions, which may result in measurement constraints, limited variable selection, and potential unmeasured confounding. We mitigated these limitations where possible by selecting validated variables, applying strict inclusion criteria, and conducting sensitivity analyses to assess the robustness of our findings. Nevertheless, some drawbacks, such as the inability to adjust for all relevant confounders or to control the timing of data collection, remain and should be considered when interpreting the results.

Despite these limitations, the current study also boasts several strengths. Foremost, in contrast to many studies relying on cross-sectional data, which are consequently constrained to a data-driven diagnostic paradigm [53–55], our study’s longitudinal design allowed us to develop prospective prediction models. Second, our dataset’s inclusion of a broad range of psychosocial, environmental, biological, and genetic predictors is relatively uncommon in the literature. Third, the unique combination of a European cohort and a less frequently investigated age range contributes to the limited pool of published studies that have used ML approaches for the prospective prediction of the (first) onset of depression/MDD. Last, to the best of our knowledge, our study is only the second of its kind to investigate the clinical utility of ML-based prospective prediction models in the realm of depression/MDD.

Future researchers could aim to integrate an even more diverse array of candidate predictors into their models. Data derived from ecological momentary assessment, imaging techniques such as magnetic resonance imaging (MRI), or large-scale electronic health records are already being used independently in the context of mental disorders. A holistic approach that amalgamates these and other varied data sources could potentially enhance predictive performance. On the other hand, the more data sources that are necessary for a prediction model, with some of these data sources being very costly (e.g., MRI), the less clinically realistic it appears to be to implement such a prediction model in practice. Further, future research should continue to explore the role of biological and genetic factors in predicting the first onset of MDD. To our knowledge, this is the first study to incorporate both types of predictors in this context using a large longitudinal cohort. Although these variables did not substantially improve predictive performance in our models, we caution against drawing firm conclusions from a single study. Further investigations are needed to clarify their potential contribution to the performance of prediction models. We fully acknowledge that large-scale cohort studies are expensive and logistically demanding, which contributes to their scarcity. Nonetheless, these datasets remain invaluable for understanding complex, long-term outcomes. To complement this, future research could also benefit from the collection of primary data specifically designed to address prediction of MDD onset, enabling more precise control over variable selection, measurement timing, and potential confounders. Finally, studies that examine changes in risk factors over time, rather than relying solely on baseline measures, may offer deeper insight into the dynamic nature of MDD development.

In conclusion, the efficacy of ML-based prediction models appears promising but has much room to improve over time. It will be intriguing to observe how these emerging methodologies will aid in enhancing the prevention and treatment of mental disorders such as MDD.

## Electronic supplementary material

Below is the link to the electronic supplementary material.

Supplementary Material 1

## Data Availability

The data of the CoLaus|PsyCoLaus study used in this article cannot be fully shared as they contain potentially sensitive personal information on participants. According to the Ethics Committee for Research of the Canton of Vaud, sharing these data would be a violation of the Swiss legislation on privacy protection. However, coded individual-level data that do not allow researchers to identify participants are available upon request to researchers who meet the criteria for data sharing of the CoLaus|PsyCoLaus Datacenter (CHUV, Lausanne, Switzerland). Any researcher affiliated with a public or private research institution who complies with the CoLaus|PsyCoLaus standards can submit a research application to research.colaus@chuv.ch or research.psycolaus@chuv.ch. Proposals requiring baseline data only will be evaluated by the baseline (local) Scientific Committee (SC) of the CoLaus and PsyCoLaus studies. Proposals requiring follow-up data will be evaluated by the follow-up (multicentric) SC of the CoLaus|PsyCoLaus cohort study. Detailed instructions for gaining access to the CoLaus|PsyCoLaus data used in this study are available at www.colaus-psycolaus.ch/professionals/how-to-collaborate/.

## References

[1] Wittchen HU, Jacobi F, Rehm J, Gustavsson A, Svensson M, Jonsson B, Olesen J, Allgulander C, Alonso J, Faravelli C, Fratiglioni L, Jennum P, Lieb R, Maercker A, van Os J, Preisig M, Salvador-Carulla L, Simon R, Steinhausen HC (2011) The size and burden of mental disorders and other disorders of the brain in Europe 2010. Eur Neuropsychopharmacol 21(9):655–679. 10.1016/j.euroneuro.2011.07.018 21896369 10.1016/j.euroneuro.2011.07.018

[2] American Psychiatric Association (2013) Diagnostic and statistical manual of mental disorders: DSM-5. Diagnostic and statistical manual of mental disorders: DSM-5, 5th edn. American Psychiatric Publishing, Inc. xliv, Arlington, VA, US, pp 947–xliv. 10.1176/appi.books.9780890425596

[3] Otte C, Gold SM, Penninx BW, Pariante CM, Etkin A, Fava M, Mohr DC, Schatzberg AF (2016) Major depressive disorder. Nat Rev Dis Primers 2:16065. 10.1038/nrdp.2016.65 27629598 10.1038/nrdp.2016.65

[4] Garcia-Toro M, Aguirre I (2007) Biopsychosocial model in depression revisited. Med Hypotheses 68(3) 683– 91. 10.1016/j.mehy.2006.02.049 10.1016/j.mehy.2006.02.049 17140747

[5] De Carlo V, Calati R, Serretti A (2016) Socio-demographic and clinical predictors of non-response/non-remission in treatment resistant depressed patients: A systematic review. Psychiatry Res 240:421–430. 10.1016/j.psychres.2016.04.034 27155594 10.1016/j.psychres.2016.04.034

[6] Kohler CA, Evangelou E, Stubbs B, Solmi M, Veronese N, Belbasis L, Bortolato B, Melo MCA, Coelho CA, Fernandes BS, Olfson M, Ioannidis JPA, Carvalho AF (2018) Mapping risk factors for depression across the lifespan: an umbrella review of evidence from meta-analyses and Mendelian randomization studies. J Psychiatr Res 103:189–207. 10.1016/j.jpsychires.2018.05.020 29886003 10.1016/j.jpsychires.2018.05.020

[7] Firmann M, Mayor V, Vidal PM, Bochud M, Pecoud A, Hayoz D, Paccaud F, Preisig M, Song KS, Yuan X, Danoff TM, Stirnadel HA, Waterworth D, Mooser V, Waeber G, Vollenweider P (2008) The CoLaus study: a population-based study to investigate the epidemiology and genetic determinants of cardiovascular risk factors and metabolic syndrome. BMC Cardiovasc Disord 8:6. 10.1186/1471-2261-8-6 18366642 10.1186/1471-2261-8-6 PMC2311269

[8] Preisig M, Waeber G, Vollenweider P, Bovet P, Rothen S, Vandeleur C, Guex P, Middleton L, Waterworth D, Mooser V, Tozzi F, Muglia P (2009) The PsyCoLaus study: methodology and characteristics of the sample of a population-based survey on psychiatric disorders and their association with genetic and cardiovascular risk factors. BMC Psychiatry 9:9. 10.1186/1471-244X-9-9 19292899 10.1186/1471-244X-9-9 PMC2667506

[9] Rudaz DA, Vandeleur CL, Gebreab SZ, Gholam-Rezaee M, Strippoli MF, Lasserre AM, Glaus J, Castelao E, Pistis G, von Kanel R, Marques-Vidal P, Waeber G, Vollenweider P, Preisig M (2017) Partially distinct combinations of psychological, metabolic and inflammatory risk factors are prospectively associated with the onset of the subtypes of major depressive disorder in midlife. J Affect Disord 222:195–203. 10.1016/j.jad.2017.07.016 28710953 10.1016/j.jad.2017.07.016

[10] Lieb R, Wittchen H-U, Höfler M, Fuetsch M, Stein MB, Merikangas KR (2000) Parental psychopathology, parenting styles, and the risk of social phobia in offspring: a prospective-longitudinal community study. Arch Gen Psychiatry 57(9):859–866 10986549 10.1001/archpsyc.57.9.859

[11] Patel JS, Berntson J, Polanka BM, Stewart JC (2018) Cardiovascular risk factors as differential predictors of incident atypical and typical major depressive disorder in US adults. Psychosom Med 80(6):508–514. 10.1097/PSY.0000000000000583 29595710 10.1097/PSY.0000000000000583 PMC6023772

[12] Bennett D, Silverstein SM, Niv Y (2019) The two cultures of computational psychiatry. JAMA Psychiatry 76(6):563–564 31017638 10.1001/jamapsychiatry.2019.0231 PMC8813275

[13] van Zoonen K, Buntrock C, Ebert DD, Smit F, Reynolds III, Beekman CF, A.T., and, Cuijpers P (2014) Preventing the onset of major depressive disorder: a meta-analytic review of psychological interventions. Int J Epidemiol 43(2):318–329 24760873 10.1093/ije/dyt175 PMC4023317

[14] Merry SN, Hetrick SE, Cox GR, Brudevold-Iversen T, Bir JJ, McDowell H (2012) Cochrane review: psychological and educational interventions for preventing depression in children and adolescents. Evidence‐Based Child Health: Cochrane Rev J 7(5):1409–1685 10.1002/14651858.CD003380.pub3 22161377

[15] Cuijpers P, Smit F, Patel V, Dias A, Li J, Reynolds CF III (2015) Prevention of depressive disorders in older adults: an overview. PsyCh J 4(1):3–10 26263525 10.1002/pchj.86

[16] Bzdok D, Meyer-Lindenberg A (2018) Machine learning for precision psychiatry: opportunities and challenges. Biol Psychiatry Cogn Neurosci Neuroimaging 3(3):223–230. 10.1016/j.bpsc.2017.11.007 29486863 10.1016/j.bpsc.2017.11.007

[17] Studerus E, Ramyead A, Riecher-Rossler A (2017) Prediction of transition to psychosis in patients with a clinical high risk for psychosis: a systematic review of methodology and reporting. Psychol Med 47(7):1163–1178. 10.1017/S0033291716003494 28091343 10.1017/S0033291716003494

[18] Dwyer DB, Falkai P, Koutsouleris N (2018) Machine learning approaches for clinical psychology and psychiatry. Annu Rev Clin Psychol 14:91–118. 10.1146/annurev-clinpsy-032816-045037 29401044 10.1146/annurev-clinpsy-032816-045037

[19] Šimundić A-M (2009) Measures of diagnostic accuracy: basic definitions. Ejifcc 19(4):203 27683318 PMC4975285

[20] Brathwaite R, Rocha TB, Kieling C, Gautam K, Koirala S, Mondelli V, Kohrt B, Fisher HL (2021) Predicting the risk of depression among adolescents in Nepal using a model developed in brazil: the IDEA project. Eur Child Adolesc Psychiatry 30(2):213–223. 10.1007/s00787-020-01505-8 32162056 10.1007/s00787-020-01505-8 PMC7486232

[21] Sampson L, Jiang T, Gradus JL, Cabral HJ, Rosellini AJ, Calabrese JR, Cohen GH, Fink DS, King AP, Liberzon I, Galea S (2021) A machine learning approach to predicting New-onset depression in a military population. Psychiatr Res Clin Pract 3(3):115–122. 10.1176/appi.prcp.20200031 34734165 10.1176/appi.prcp.20200031 PMC8562467

[22] Gu SC, Zhou J, Yuan CX, Ye Q (2020) Personalized prediction of depression in patients with newly diagnosed parkinson’s disease: A prospective cohort study. J Affect Disord 268:118–126. 10.1016/j.jad.2020.02.046 32158001 10.1016/j.jad.2020.02.046

[23] Librenza-Garcia D, Passos IC, Feiten JG, Lotufo PA, Goulart AC, de Souza Santos I, Viana MC, Bensenor IM, Brunoni AR (2021) Prediction of depression cases, incidence, and chronicity in a large occupational cohort using machine learning techniques: an analysis of the ELSA-Brasil study. Psychol Med 51(16):2895–2903. 10.1017/S0033291720001579 32493535 10.1017/S0033291720001579

[24] Rosellini AJ, Liu S, Anderson GN, Sbi S, Tung ES, Knyazhanskaya E (2020) Developing algorithms to predict adult onset internalizing disorders: an ensemble learning approach. J Psychiatr Res 121:189–196. 10.1016/j.jpsychires.2019.12.006 31864158 10.1016/j.jpsychires.2019.12.006 PMC7027595

[25] Hawes MT, Schwartz HA, Son Y, Klein DN (2022) Predicting adolescent depression and anxiety from multi-wave longitudinal data using machine learning. Psychol Med 1–7. 10.1017/S0033291722003452 10.1017/S0033291722003452 36377499

[26] Cho SE, Geem ZW, Na KS (2021) Predicting depression in community dwellers using a machine learning algorithm. Diagnostics 11(8):1429. 10.3390/diagnostics11081429 10.3390/diagnostics11081429 PMC8394838 34441363

[27] Lin S, Wu Y, Fang Y (2021) Comparison of regression and machine learning methods in depression forecasting among Home-Based elderly chinese: A community based study. Front Psychiatry 12:764806. 10.3389/fpsyt.2021.764806 35111085 10.3389/fpsyt.2021.764806 PMC8801448

[28] Su D, Zhang X, He K, Chen Y (2021) Use of machine learning approach to predict depression in the elderly in china: A longitudinal study. J Affect Disord 282:289–298. 10.1016/j.jad.2020.12.160 33418381 10.1016/j.jad.2020.12.160

[29] Lang M, Binder M, Richter J, Schratz P, Pfisterer F, Coors S, Au Q, Casalicchio G, Kotthoff L, Bischl B (2019) mlr3: A modern object-oriented machine learning framework in R. J Open Source Softw 4(44):p1903. 10.21105/joss.01903

[30] Core Team R (2022) R: A Language and environment for statistical computing. R Foundation for Statistical Computing: Vienna, Austria

[31] Team P (2022) *RStudio: Integrated Development Environment for R*. Posit Software, PBC: Boston, MA

[32] Friedman J, Hastie T, Tibshirani R (2010) Regularization paths for generalized linear models via coordinate descent. J Stat Softw 33(1):1–22 20808728 PMC2929880

[33] Wright MN, Ziegler A (2017) Ranger: A fast implementation of random forests for high dimensional data in C + + and R. J Stat Softw 77(1):1–17. 10.18637/jss.v077.i01

[34] Chen T, Guestrin C (2016) *XGBoost: A Scalable Tree Boosting System*, in *Proceedings of the 22nd ACM SIGKDD International Conference on Knowledge Discovery and Data Mining*. Association for Computing Machinery: San Francisco, California, USA. pp. 785–794

[35] Kuhn M, Vaughan D (2022) *probably: Tools for Post-Processing Class Probability Estimates* https://github.com/tidymodels/probably

[36] Krstajic D, Buturovic LJ, Leahy DE, Thomas S (2014) Cross-validation pitfalls when selecting and assessing regression and classification models. J Cheminform 6(1):1–15 24678909 10.1186/1758-2946-6-10 PMC3994246

[37] Simon R (2007) *Resampling strategies for model assessment and Selection*, in *Fundamentals of data mining in genomics and proteomics*. Springer US, Boston, MA, pp 173–186. W. Dubitzky, M. Granzow, and D. Berrar, Editors

[38] Bischl B, Binder M, Lang M, Pielok T, Richter J, Coors S, Thomas J, Ullmann T, Becker M, Boulesteix AL, Deng D, Lindauer M (2023) Hyperparameter optimization: Foundations, algorithms, best practices, and open challenges. WIREs Data Mining and Knowledge Discovery, 13(2):e1484. 10.1002/widm.1484

[39] Ding Y, Simonoff JS (2010) An investigation of missing data methods for classification trees applied to binary response data. J Mach Learn Res 11(6):131–170. http://jmlr.org/papers/v11/ding10a.html

[40] Binder M, Pfisterer F, Lang M, Schneider L, Kotthoff L, Bischl B (2021) mlr3pipelines - Flexible machine learning pipelines in R. J Mach Learn Res 22(184):1–7

[41] Molnar C, Casalicchio G, Bischl B (2018) Iml: an R package for interpretable machine learning. J Open Source Softw 3(26):786

[42] van Buuren S, Groothuis-Oudshoorn K (2011) mice: multivariate imputation by Chained equations in R. J Stat Soft 45(3):1–67. 10.18637/jss.v045.i03

[43] Sjoberg D (2022) *dcurves: Decision Curve Analysis for Model Evaluation* https://github.com/ddsjoberg/dcurves

[44] Vickers AJ, Elkin EB (2006) Decision curve analysis: a novel method for evaluating prediction models. Med Decis Making 26(6):565–574 17099194 10.1177/0272989X06295361 PMC2577036

[45] Vickers AJ, van Calster B, Steyerberg EW (2019) A simple, step-by-step guide to interpreting decision curve analysis. Diagn Prognostic Res 3(1):1–8 10.1186/s41512-019-0064-7 PMC6777022 31592444

[46] Kessler RC, Bauer MS, Bishop TM, Bossarte RM, Castro VM, Demler OV, Gildea SM, Goulet JL, King AJ, Kennedy CJ (2023) Evaluation of a model to target high-risk psychiatric inpatients for an intensive postdischarge suicide prevention intervention. JAMA Psychiatry 80(3):230–240 36652267 10.1001/jamapsychiatry.2022.4634 PMC9857842

[47] Van Calster B, Vickers AJ (2015) Calibration of risk prediction models: impact on decision-analytic performance. Med Decis Mak 35(2):162–169. 10.1177/0272989X14547233 10.1177/0272989X14547233 25155798

[48] Van Calster B, Wynants L, Verbeek JF, Verbakel JY, Christodoulou E, Vickers AJ, Roobol MJ, Steyerberg EW (2018) Reporting and interpreting decision curve analysis: a guide for investigators. Eur Urol 74(6):796–804 30241973 10.1016/j.eururo.2018.08.038 PMC6261531

[49] Huang Y, Li W, Macheret F, Gabriel RA, Ohno-Machado L (2020) A tutorial on calibration measurements and calibration models for clinical prediction models. J Am Med Inform Assoc 27(4):621–633 32106284 10.1093/jamia/ocz228 PMC7075534

[50] Karvelis P, Paulus MP, Diaconescu AO (2023) Individual differences in computational psychiatry: A review of current challenges. Neurosci Biobehav Rev 148:105137. 10.1016/j.neubiorev.2023.105137 36940888 10.1016/j.neubiorev.2023.105137

[51] Coughlin SS (1990) Recall bias in epidemiologic studies. J Clin Epidemiol 43(1):87–91 2319285 10.1016/0895-4356(90)90060-3

[52] Hakulinen C, Elovainio M, Pulkki-Råback L, Virtanen M, Kivimäki M, Jokela M (2015) Personality and depressive symptoms: individual participant meta‐analysis of 10 cohort studies. Depress Anxiety 32(7):461–470 26014798 10.1002/da.22376 PMC4605994

[53] Aleem S, Huda Nu, Amin R, Khalid S, Alshamrani SS, Alshehri A (2022) Machine learning algorithms for depression: diagnosis, insights, and research directions. Electronics 11(7):1111

[54] Gil M, Kim SS, Min EJ (2022) Machine learning models for predicting risk of depression in Korean college students: identifying family and individual factors. Front Public Health 10:1023010. 10.3389/fpubh.2022.1023010 36466485 10.3389/fpubh.2022.1023010 PMC9714606

[55] Handing EP, Strobl C, Jiao Y, Feliciano L, Aichele S (2022) Predictors of depression among middle-aged and older men and women in europe: A machine learning approach. Lancet Reg Health-Europe 18:100391 10.1016/j.lanepe.2022.100391 PMC9065918 35519235

