# Supplementary material for: Prospective prediction of first onset of major depressive disorder in midlife using machine learning
Source: Soc Psychiatry Psychiatr Epidemiol. 2025 Jun 18;60(10):2387–400. doi: 10.1007/s00127-025-02942-z (PMC12449384; doi:10.1007/s00127-025-02942-z)
Supplement: Supplementary file 1 — Supplementary Material 1 [file 127_2025_2942_MOESM1_ESM.docx]

**Table S1**

*Studies prospectively predicting first onset of depression using machine learning: Study characteristics*

| Citation | Location | Population | Age (years) | Study type | Sample size | Depression cases | Prediction window | Outcome | Predictor domain | Assessment instruments | Model |
| --- | --- | --- | --- | --- | --- | --- | --- | --- | --- | --- | --- |
| Brathwaite et al. (2021) | Nepal | Child soldiers | <18 | Adolescent cohort | 126 | 25 | 4 years | DSRS for children | Soc, env | Questionnaires | Penalized LR |
| Cho et al. (2021) | Korea | Community dwellers | 50+ | National survey | 9,488 | 584 | 2 years | PHQ-9 | Soc, econ, clin | Questionnaires, blood, urine | Penalized LR |
| Gu et al. (2020) | Multiple | Parkinson’s patients | 30+ | Prospective cohort | 312 | 36 | 2 years | GDS-15 | Clin | Questionnaires | xgb, LR |
| Hawes et al. (2022) | USA | Children and adolescents | 3–15 | Community sample | 374 | 22 at 12,  ? at 15 | 3, 6, 9, 12 years | SADS | Clin, pers, env, bio, neuro, parental | Interviews, questionnaires, saliva, behavioral & neural | Penalized LR |
| Librenza-Garcia et al. (2020) | Brazil | Civil servants | 35–74 | Occupational cohort | 13,922 | 499 | 2–4 years | CIS-R | Soc, clin | Questionnaires, interviews | Penalized LR |
| Lin et al. (2021) | China | Home-based elderly | 60+ | Representative cohort | 3,570 | 976 | 2 years | CESD-10 | Soc, econ, health | Questionnaires | LR, penalized LR, RF |
| Rosellini et al. (2020) | USA | General population | 18+ | Representative household survey | 27,769 | 1,707 | 2–4 years | AUDADIS-IV | Soc, health, mental, pers, env | Questionnaires | ensembles |
| Sampson et al. (2021) | USA | National guard members | 25+ | Cohort | 2,249 | 367 | 5 years | PHQ-9 + *DSM-IV* criteria | Soc, military, Health, env, stress | Questionnaires | CTrees, RF |
| Su et al. (2021) | China | Elderly | 65+ | Survey | 1,538 | 289 | 2 years | 2 questions | Soc, health, chronic | Questionnaires | LR, penalized LR, RF, GB, SVM, DNN |

*Note*. DSRS = Birleson Depression Self-Rating Scale; PHQ = Patient Health Questionnaire; GDS = Geriatric Depression Scale; SADS = Schedule for Affective Disorders and Schizophrenia; CIS-R = Clinical Interview Schedule–Revised; CESD = Center for Epidemiologic Studies Depression Scale; AUDADIS = Alcohol Use Disorder and Associated Disabilities Interview Schedule; *DSM-IV* = *Diagnostic and statistical manual of mental disorders* (4th ed.); soc = sociodemographic; env = environmental; econ = economic; pers = personality; bio = biological; neuro = neurological; LR = logistic regression; xgb = XGBoost; RF = random forests; CTrees = classification trees; GB = gradient boosting; SVM = support vector machines; DNN = deep neural networks.

**Table S2**

*Studies prospectively predicting first onset of depression using machine learning: Performance estimates*

| Citation | PRAUC | AUC | ACC | TPR / Sensitivity | TNR / Specificity | PPV / Precision | NPV | BRIER | Resampling procedure | Nested resampling | Net benefit |
| --- | --- | --- | --- | --- | --- | --- | --- | --- | --- | --- | --- |
| Brathwaite (2021) | – | 0.73 / 0.83 | – | – | – | – | – | 0.18 / 0.14 | ext / none | – | – |
| Cho (2021) | – | 0.9 | 0.82 | 0.82 | 0.82 | 0.23 | 0.99 | – | 10CV | – | – |
| Gu (2020) | – | 0.89 / 0.94 | – | 0.81 / 0.92 | 0.83 / 0.86 | – | – | – | 10CV | – | – |
| Hawes (2022) | – | 0.55–0.75 | – | - | - | – | – | – | 10CV | – | – |
| Librenza-Garcia (2020) | – | 0.71 | 0.68 | 0.61 | 0.75 | 0.07 | 0.98 | – | 10RepCV | – | – |
| Lin (2021) | – | 0.77–0.8 | – | 0.29–0.4 | – | 0.64–0.66 | – | 0.16 | 10RepCV | Yes | – |
| Rosellini (2020) | – | 0.76 | – | 0.69 | – | 0.14 | – | 0.02–0.05 | 10CV | – | – |
| Sampson (2021) | – | 0.67 | 0.73 / 0.68 | 0.47 / 0.46 | 0.77 / 0.75 | – | – | – | 10CV | – | – |
| Su (2021) | – | 0.59–0.63 | 0.48–0.76 | 0.43–0.75 | 0.43–0.83 | 0.21–0.33 | 0.87–0.9 | – | 10CV | – | Yes |

*Note*. PRAUC = Area under the precision-recall curve; AUC = area under the receiver operating characteristic curve; ACC = accuracy; TPR = true positive rate; TNR = true negative rate; PPV = positive predictive value; NPV = negative predictive value; BRIER = binary Brier score; ext = external; CV = cross-validation; RepCV = repeated cross-validation.

**Table S3**

*Tuned hyperparameters (HPs), corresponding search spaces, and mean HP values*

| **Hyperparameter** | **Range** | **Default** | **MeanHP value** |
| --- | --- | --- | --- |
| **Logistic regression** | – | – | – |
| **Elastic net** |  |  |  |
| s | [-12, 12] | 1 | -3.80 |
| alpha_el | [0, 1] | 1 | 0.55 |
| **Random forests** |  |  |  |
| mtry | {1, ..., p} | \|√p\| | 0.33 |
| replace | [FALSE, TRUE] | TRUE | 0.48 |
| sample_fraction | [0.1, 1] | 1 (if replace=TRUE) or 0.632 | 0.54 |
| num_trees | {1, ..., 2000} | 500 | 1078 |
| **XGBoost** |  |  |  |
| eta | [-4, 0] | 0.3 | -5.35 |
| nrounds | {1, ..., 5000} | - | 2496 |
| max_depth | {1, ..., 20} | 6 | 8.89 |
| colsample_bytree | [0.1, 1] | 1 | 0.51 |
| colsample_bylevel | [0.1, 1] | 1 | 0.47 |
| lambda | [-10, 10] | 1 | 0.92 |
| alpha_xgb | [-10, 10] | 0 | -1.20 |
| subsample | [0.1, 1] | 1 | 0.64 |

*Note*. s = controls regularization; alpha_el = determines mixture of the lasso and ridge penalties; mtry = number of features considered as candidate splitting variables at each split; replace = controls if observations are drawn with or without replacement; sample_fraction = proportion of randomly drawn observations; num_trees = number of trees; eta = learning rate; nrounds = number of boosting iterations; max_depth = maximum depth of a tree; colsample_bytree = subsample ratio of columns for each tree; colsample_bylevel = subsample ratio of columns for each depth level; lambda = L2 regularization term on weights; alpha_xgb = L1 regularization term on weights; subsample = subsample ratio of the training instances, Mean HP value = mean hyperparameter value over 100 inner resample iterations.

**Fig. S1**

*Aggregated model performance metrics based on nested cross-validation procedure*

**
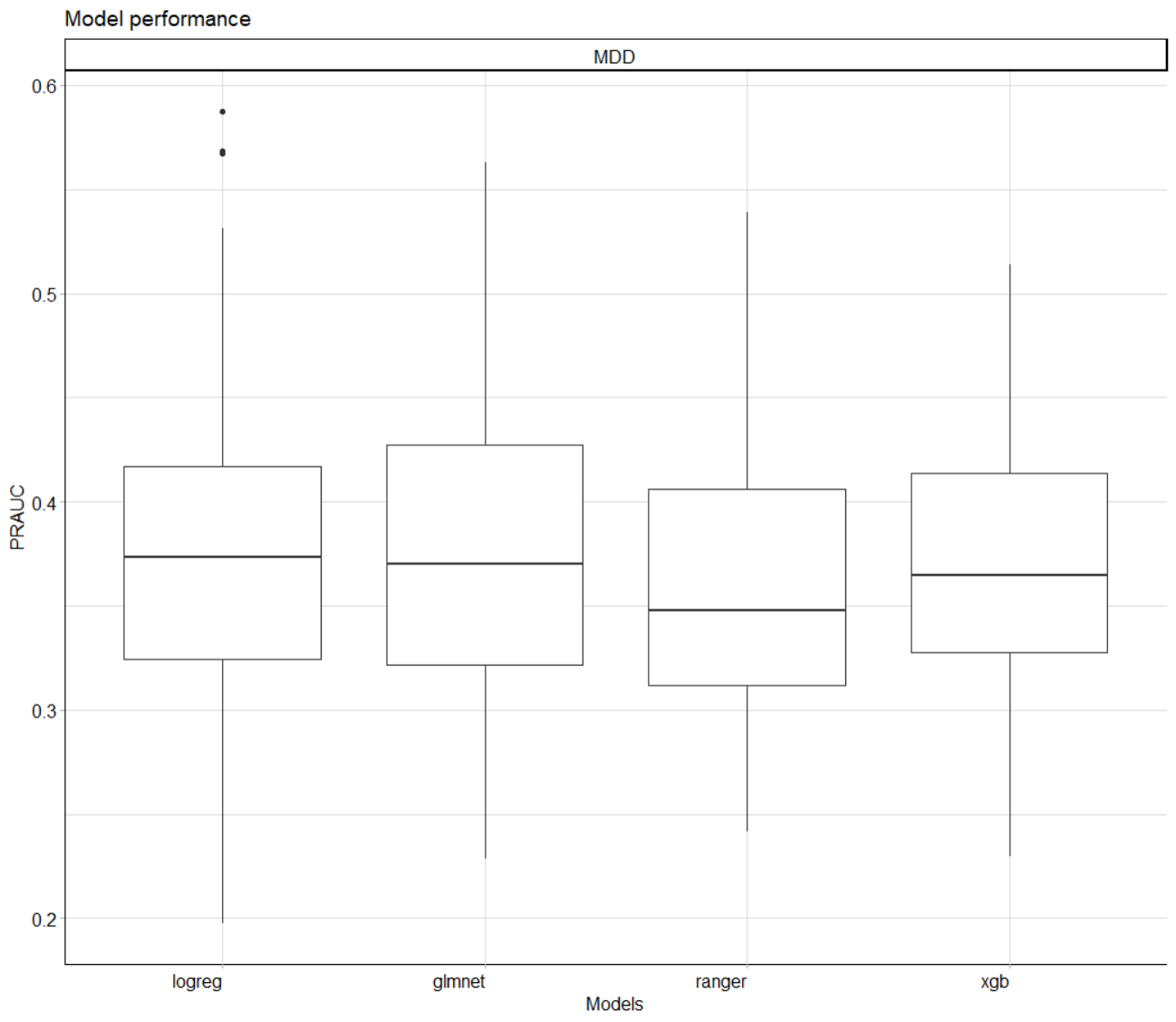
**

*Note.* MDD = major depressive disorder; PRAUC = area under the precision-recall curve; logreg = logistic regression; glmnet = elastic net; ranger = random forests; xgb = XGBoost. Higher PRAUC values indicate better discriminative model performance.

**Fig. S2**

*Aggregated model performance metrics based on nested cross-validation procedure*

**
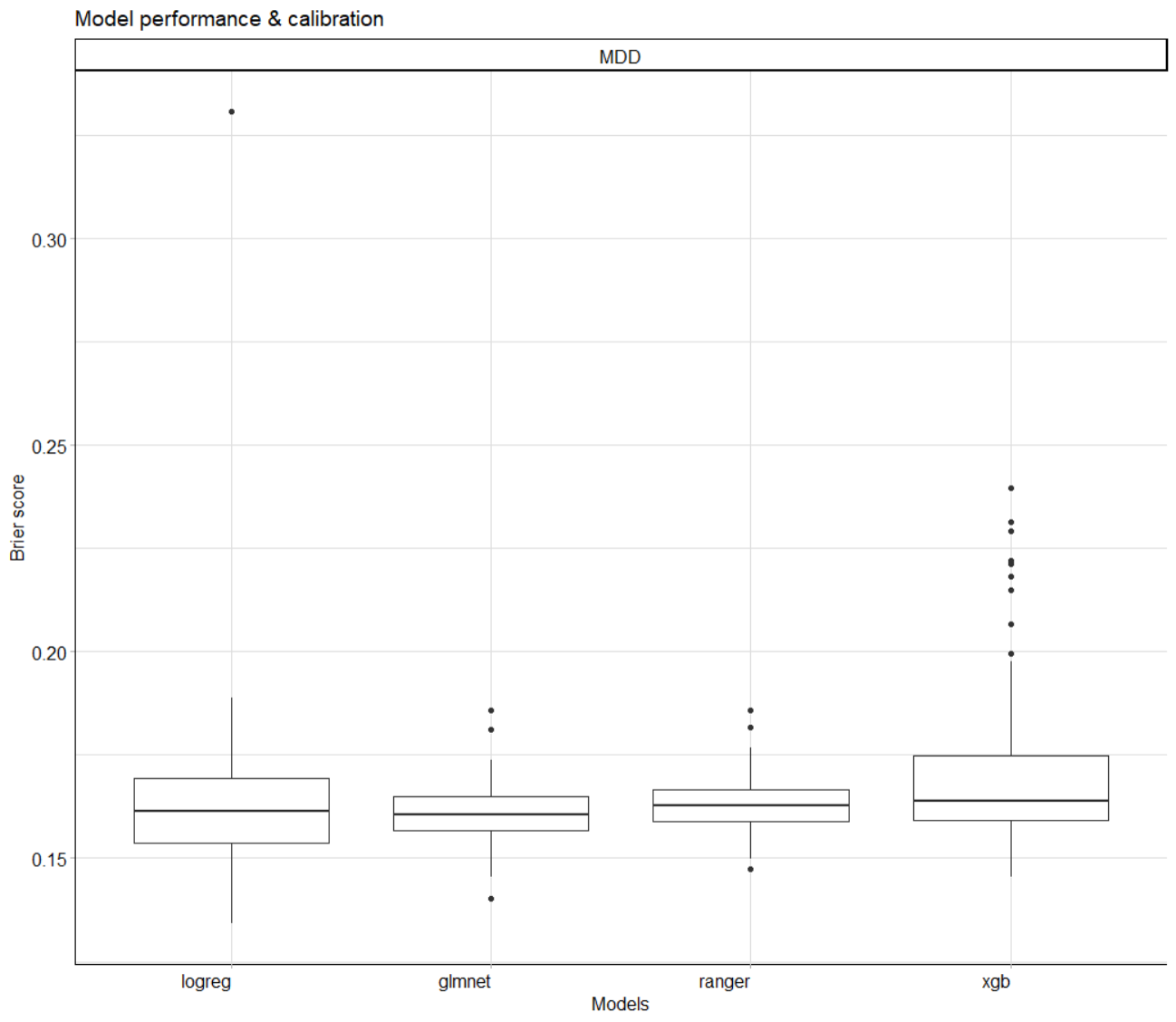
**

*Note.* MDD = major depressive disorder; logreg = logistic regression; glmnet = elastic net; ranger = random forests; xgb = XGBoost. Lower BRIER scores indicate better discriminative performance and calibration.

**SM1: Measures**

Participants were interviewed by master-level psychologists who were trained over a 1- to 2-month period. Diagnostic information on mental disorders was collected at baseline and each follow-up using the French version of the semi-structured Diagnostic Interview for Genetic Studies (DIGS; [1, 2]. The French version of the DIGS has undergone extensive testing prior to the initiation of the present study and has revealed excellent inter-rater and fair to good test-retest reliability for major mood (e.g. kappa coefficients for inter-rater and test-retest reliability for MD of kappa = 0.94 and 0.67, respectively) (Preisig, Fenton, Matthey, Berney, & Ferrero, 1999) and substance use disorders (Berney, Preisig, Matthey, Ferrero, & Fenton, 2002). The DIGS was completed with anxiety disorder sections of the Schedule for Affective Disorders and Schizophrenia–Lifetime Version [3], which also revealed excellent inter-rater and fair-to-good test-retest reliability [4]. Lifetime diagnoses of mental disorders (MDD, dysthymia, anxiety disorders, and substance use disorders) were assigned according to the *DSM-IV* [5]. The lifetime diagnosis for the category Other Specified Depressive Disorders (OSDD) was assigned according to the *DSM-5* (American Psychiatric Association, 2013). The DIGS also collects information on sociodemographic characteristics (sex, age, marital status, and socioeconomic status). Socioeconomic status was defined according to the Hollingshead scale [6].

Childhood stressful life events before the age of 18 years encompassing (1) accident or severe catastrophe, (2) violent crime, (3) active combat or war, (4) witnessing trauma to others, and (5) exposure to sexual trauma, including rape, sexual abuse, and exhibitionism were assessed in the posttraumatic stress disorder section of the DIGS. Events during adulthood were elicited using the life-event interview of Amiel-Lebigre [7], which covers 52 potentially stressful life-events as well as their timing and their negative affective impact ranging from 0 to 100, allowing us to compute cumulative severity scores [8].

Information on smoking and physical activity was collected through a standardized interview. Nicotine consumption entailed a current daily consumption of at least 10 cigarettes for at least 2 months. Participants were considered to be physically inactive if they reported engaging in physical activity for less than 20 min twice a week.

Family history information on MDD was collected using the Family History–Research Diagnostic Criteria (FH-RDC; [9]. The validity of the French version of the FH-RDC has previously been established through the assessment of agreement between diagnoses relying on family history reports and direct interviews, including MDD [10].

Neuroticism, extraversion, and psychoticism were assessed using the French version of the Eysenck Personality Questionnaire [11]. The originator of this instrument reported Cronbach’s α coefficients of 0.78 to 0.87 for neuroticism, 0.72 to 0.82 for extraversion, and 0.60 to 0.72 for psychoticism, using three different French samples [12].

Coping strategies were evaluated using the French version [13] of the coping section of the Euronet questionnaire [14], which includes the factors emotion-focused coping, help-seeking behaviors, and problem-focused coping according to principal component analysis [15]. The standardized Cronbach’s α coefficients for these dimensions were 0.65, 0.69, and 0.44, respectively [15]. As emotion-focused coping was highly correlated with neuroticism (r = 0.63; p < 0.0001), we could not include it in our analyses.

Information on smoking and physical activity was collected through a standardized interview. Nicotine consumption entailed a current daily consumption of at least 10 cigarettes for at least 2 months. Participants were considered to be physically inactive if they reported engaging in physical activity for less than 20 min twice a week.

Physical measures taken at baseline are described in detail elsewhere [16]. The assessment included weight and height to determine body mass index. Blood pressure was measured three times on the left arm after at least a 10-min rest in the seated position. The mean of the last two measures was used. Venous blood samples were drawn after an overnight fast to measure the levels of glucose, high-density lipoprotein (HDL) cholesterol, low-density lipoprotein (LDL) cholesterol, triglycerides, interleukin-1β (IL-1β), interleukin-6 (IL-6), tumor necrosis factor-α (TNF-α), and high-sensitivity C-reactive protein (hs-CRP). To adjust for the effects of medication, values of HDL cholesterol, triglycerides, and systolic blood pressure (SBP) for treated subjects were assigned according to documented mean changes under medication [17]. For subjects using fibrates, 0.10 mmol/liter was subtracted from HDL cholesterol and 0.67 mmol/liter was added to triglycerides, and for those using antihypertensive drugs 10 mmHg was added to the SBP. We assessed hs-CRP using immunoassay and latex HS (IMMULITE 1000-High, Diagnostic Products Corporation, Los Angeles, CA, USA), with maximum intra- and interbatch coefficients of variation of 1.3% and 4.6%, respectively [16]. For cytokine measurements, serum samples were stored at -80 °C before assessment and sent on dry ice to the laboratory. Cytokine concentrations were measured using a multiplexed particle-based flow cytometric cytokine assay [18]. The lower detection limit (LOD) for IL-1β, IL-6, and TNF-α was 0.2 pg/ml. For concentrations below the LOD (37.5% for IL-1β, 8.2% for IL-6, 0.7% for TNF-α), a value of 0.1 pg/ml was assigned. Good agreement between signal and cytokine was found within the assay range (R^2^≥0.99). Intra- and interassay coefficients of variation were 15% and 16.7% for IL-1β, 16.9% and 16.1% for IL-6, and 12.5% and 13.5% for TNF-α, respectively. Repeated measurements were conducted in 80 subjects randomly drawn from the initial sample; Spearman rank correlations between duplicate measurements were 0.914, 0.961, and 0.891 for IL-1β, IL-6, and TNF-α (all p < 0.001). Diabetes was diagnosed when fasting blood glucose ≥7 mmol/l was measured or when the subject was treated for diabetes.

Genome-wide genotyping was performed using the Affymetrix 500K single nucleotide polymorphism (SNP) array. Nuclear DNA was extracted from whole blood of all participants. Genotypes were called using BRLMM (http://www.affymetrix.com/support/technical/whitepapers/brlmm_whitepap). Duplicate individuals, and first- and second-degree relatives, were identified and then removed by computing estimated pairwise genomic kinship coefficients, using kinship-based inference [19]. Subjects were excluded from the analysis in case of inconsistency between sex and genetic data, a genotype call rate of less than 90%, or inconsistencies of genotyping results in duplicate samples. Quality control for SNPs was performed using the following criteria: monomorphic (or with minor allele frequency [MAF] < 1%), call rates less than 90%, and deviation from the Hardy–Weinberg equilibrium (p < 1×10^-6^). Phased haplotypes were generated using SHAPEIT2 [20, 21]. Imputation was performed using minimac3 [22] and the Haplotype Reference Consortium version r1.1 [23], hosted on the Michigan Imputation Server [22]. We used imputed allele dosages for all SNPs to avoid genotyping missingness. A MAF > 1% and an imputation quality Rsq > 0.3 was required for the inclusion of the variant in further analyses.

Polygenic risk scores (PRSs) were derived using PRSice-2 software [24] and effect sizes from a large MDD genome-wide association study (GWAS) meta-analysis of the 33 cohorts of the Psychiatric Genomics Consortium (excluding UK Biobank and 23andMe data) as described in Wray et al. [25] and the broad depression phenotype in the full release of the UK Biobank as described in Howard et al. [26]. The total number of individuals in this data is 500,199 (170,756 cases and 329,443 controls). Linkage disequilibrium clumping was performed to retain only data for independent SNPs (r^2^ < 0.1, 250 kb window). In a preliminary analysis, we compared 7 PRSs based on GWAS p-value thresholds between 5e^-8^ (only genome-wide significant SNPs) and 1 (all SNPs), as well as one PRS calculated using the expression quantitative trait loci for five of the six genes (WWC3 was excluded because it falls on the X chromosome, which is not included in the MDD GWAS) associated with MDD reported in Zhao et al. [27]. The PRS based on the GWAS p-value threshold of ≤ 0.05 performed best in terms of the area under the receiver operating characteristic curve (as also shown by Wray et al. [25] and was used in all prediction models reported in this manuscript.

**SM2: Robustness check: Analyses without imputation**

**Listwise deletion**

Listwise deletion was performed for all individuals with any missing values and reduced the original sample size (n = 1350) to n = 885.

**Descriptive statistics**

A total of 192 participants (21.69%) developed first onset of major depressive disorder (MDD) during the follow-up period. Characteristics of participants at baseline are reported in Table S4.

**Table S4**

*Characteristics of participants at baseline*

| **Variable** | **Mean/Median/n** | **SD/IQR/%** |
| --- | --- | --- |
| **Sociodemographic characteristics** |  |  |
| Female, n (%) | 400 | (45.2) |
| Age (years), mean (SD) | 52.1 | (8.8) |
| Socioeconomic status ^a^, mean (SD) | 3.49 | (1.24) |
| Married, n (%) | 593 | (67) |
| **Family history** |  |  |
| Relatives with MDD, n (%) | 330 | (37.2) |
| **Other lifetime diagnoses** |  |  |
| Dysthymia, n (%) | 31 | (3.5) |
| OSDD ^b^, n (%) | 161 | (18.1) |
| Alcohol use disorders, n (%) | 95 | (10.7) |
| Drug use disorders, n (%) | 42 | (4.75) |
| Anxiety disorders ^c^, n (%) | 113 | (12.8) |
| **Cardio-metabolic risk factors** |  |  |
| BMI (kg/m^2^), mean (SD) | 25.2 | (4.1) |
| Diabetes, n (%) | 42 | (4.8) |
| HDL-cholesterol (mmol/L) ^d^, mean (SD) | 1.65 | (0.43) |
| Triglycerides (mmol/L) ^d^, mean (SD) | 1.36 | (1.1) |
| SBP (mmHg) ^d^, mean (SD) | 128 | (18.2) |
| Interleukin-1β, median (IQR) | -0.9 | (2.8) |
| Interleukin-6, median (IQR) | 0.2 | (1.6) |
| TNF-α, median (IQR) | 1 | (0.9) |
| hs-CRP, median (IQR) | 0 | (1.5) |
| **Lifetime events** |  |  |
| Childhood traumatic events ^e^, n (%) | 45 | (5.1) |
| Adult life-event negative impact score, median (IQR) | 5.5 | (1.2) |
| **Behavioral factors** |  |  |
| Current smoking, n (%) | 200 | (22.6) |
| Physical inactivity ^f^, n (%) | 327 | (37) |
| **Personality** |  |  |
| Neuroticism, mean (SD) | 7.73 | (5) |
| Extraversion, mean (SD) | 12.3 | (4.8) |
| Psychoticism, mean (SD) | 5.71 | (3.12) |
| **Coping** |  |  |
| Problem-solving coping, mean (SD) | 7.66 | (1.76) |
| Help-seeking, mean (SD) | 4.1 | 2.5 |
| **Polygenic risk score** |  |  |
| Polygenic risk score for depression (p-value cut-off 0.05), mean (SD) | -79.2 | (1.29) |

*Note*. Descriptive statistics are presented as mean (SD) for continuous variables, median (IQR) for log-transformed continuous variables, and frequency (percentage) for categorical variables. IQR = interquartile range; MDD = major depressive disorder; BMI = body mass index; SBP = systolic blood pressure; HDL = high-density lipoprotein; TFN- α = tumor necrosis factor-α; hs-CRP = high-sensitivity C-reactive protein; OSDD = other specified depressive disorders.

^a^ A value of 3 represents a socioeconomic status of III (middle class) on the Hollingshead scale; ^b^ brief depression, recurrent brief depression, and minor depression; ^c^ panic disorder, agoraphobia, generalized anxiety disorder, and social phobia; ^d^ corrected for medication; ^e^ accident, crime, witness, war, sexual abuse, physical abuse; ^f^ less than 20 min twice a week.

**Predictive performance**

**Table S5**

*Aggregated model performance metrics based on nested cross-validation procedure*

| **Model** | **PRAUC** | **AUC** | **ACC** | **TPR** | **TNR** | **PPV** | **NPV** | **BRIER** |
| --- | --- | --- | --- | --- | --- | --- | --- | --- |
| Logistic regression | 0.40 | 0.67 | 0.79 | 0.15 | 0.96 | 0.56 | 0.80 | 0.16 |
| Elastic net | 0.41 | 0.69 | 0.79 | 0.03 | 0.99 | 0.58^a^ | 0.79 | 0.16 |
| Random forests | 0.37 | 0.65 | 0.78 | 0.02 | 0.99 | 0.53^a^ | 0.78 | 0.16 |
| XGBoost | 0.38 | 0.67 | 0.78 | 0.01 | 0.99 | 0.46^a^ | 0.79 | 0.17 |

*Note*. PRAUC = area under the precision-recall curve; AUC = area under the receiver operating characteristic curve; ACC = accuracy; TPR = true positive rate; TNR = true negative rate; PPV = positive predictive value; NPV = negative predictive value; BRIER = binary Brier score.

^a^ Mean positive predictive value for the subset of model fits that did not return “not a number” due to not classifying any individual as belonging to the positive group.

# References

1. Nurnberger, J.I., Jr., Blehar, M.C., Kaufmann, C.A., York-Cooler, C., Simpson, S.G., Harkavy-Friedman, J., Severe, J.B., Malaspina, D., and Reich, T., *Diagnostic interview for genetic studies. Rationale, unique features, and training. NIMH Genetics Initiative.* Arch Gen Psychiatry, 1994. 51(11): p. 849-59; discussion 863-4. <https://doi.org/10.1001/archpsyc.1994.03950110009002>

2. Preisig, M., Fenton, B.T., Matthey, M.L., Berney, A., and Ferrero, F., *Diagnostic interview for genetic studies (DIGS): inter-rater and test-retest reliability of the French version.* Eur Arch Psychiatry Clin Neurosci, 1999. 249(4): p. 174-9. <https://doi.org/10.1007/s004060050084>

3. Endicott, J. and Spitzer, R.L., *A diagnostic interview: the schedule for affective disorders and schizophrenia.* Arch Gen Psychiatry, 1978. 35(7): p. 837-44. <https://doi.org/10.1001/archpsyc.1978.01770310043002>

4. Leboyer, M., Maier, W., Teherani, M., Lichtermann, D., D'Amato, T., Franke, P., Lepine, J.P., Minges, J., and McGuffin, P., *The reliability of the SADS-LA in a family study setting.* Eur Arch Psychiatry Clin Neurosci, 1991. 241(3): p. 165-9. <https://doi.org/10.1007/BF02219716>

5. American Psychiatric Association, *DSM-IV-TR: Diagnostic and statistical manual of mental disorders (4th ed, text revision ed.)*, ed. T.F.o. DSM-IV. 2000, Washington, DC: American Psychiatric Association. (Errata)

6. Hollingshead, A.B., *Four factor index of social status*. 1975, New Haven, CT.

7. Amiel-Lebigre, F., Pelc, I., and Lagorce, A. *Evénements existentiels et dépression: une étude comparative de plusieurs types de déprimés*. in *Annales médico-psychologiques*. 1984.

8. Gebreab, S.Z., Vandeleur, C.L., Rudaz, D., Strippoli, M.F., Gholam-Rezaee, M., Castelao, E., Lasserre, A.M., Glaus, J., Pistis, G., Kuehner, C., von Kanel, R., Marques-Vidal, P., Vollenweider, P., and Preisig, M., *Psychosocial Stress Over the Lifespan, Psychological Factors, and Cardiometabolic Risk in the Community.* Psychosom Med, 2018. 80(7): p. 628-639. <https://doi.org/10.1097/PSY.0000000000000621>

9. Andreasen, N.C., Endicott, J., Spitzer, R.L., and Winokur, G., *The family history method using diagnostic criteria. Reliability and validity.* Arch Gen Psychiatry, 1977. 34(10): p. 1229-35. <https://doi.org/10.1001/archpsyc.1977.01770220111013>

10. Vandeleur, C.L., Rothen, S., Lustenberger, Y., Glaus, J., Castelao, E., and Preisig, M., *Inter-informant agreement and prevalence estimates for mood syndromes: direct interview vs. family history method.* J Affect Disord, 2015. 171: p. 120-7. <https://doi.org/10.1016/j.jad.2014.08.048>

11. Eysenck, H.J. and Eysenck, S.B.G., *Manual of the Eysenck Personality Questionnaire (junior & adult)*. 1975: Hodder and Stoughton Educational. (Errata)

12. Eysenck, H., Eysenck, S., Gauquelin, M., Gauquelin, F., Pascal, C., and Pascal, D., *La Structure de la Personnalite Chez des Francais Confrontee a Celle des Anglais Comparaison ‘‘Cross-Culturelle’’.* La Personnalite, 1980. 2: p. 7-29.

13. Bodmer, N.M. and Grob, A., *Bien‐ětre et contraintes d'adolescents: Une comparaison entre adolescents de familles monoparentales et de families biparentales.* International Journal of Psychology, 1996. 31(1): p. 39-48.

14. Grob, A., Bodmer, N., and Flammer, A., *Living conditions in Europe: the case of Switzerland.* University of Bern, Institute of Psychology, Bern, 1993.

15. Perrin, M., Vandeleur, C.L., Castelao, E., Rothen, S., Glaus, J., Vollenweider, P., and Preisig, M., *Determinants of the development of post-traumatic stress disorder, in the general population.* Soc Psychiatry Psychiatr Epidemiol, 2014. 49(3): p. 447-57. <https://doi.org/10.1007/s00127-013-0762-3>

16. Firmann, M., Mayor, V., Vidal, P.M., Bochud, M., Pecoud, A., Hayoz, D., Paccaud, F., Preisig, M., Song, K.S., Yuan, X., Danoff, T.M., Stirnadel, H.A., Waterworth, D., Mooser, V., Waeber, G., and Vollenweider, P., *The CoLaus study: a population-based study to investigate the epidemiology and genetic determinants of cardiovascular risk factors and metabolic syndrome.* BMC Cardiovasc Disord, 2008. 8: p. 6. <https://doi.org/10.1186/1471-2261-8-6>

17. Licht, C.M., de Geus, E.J., and Penninx, B.W., *Dysregulation of the autonomic nervous system predicts the development of the metabolic syndrome.* The Journal of Clinical Endocrinology & Metabolism, 2013. 98(6): p. 2484-2493.

18. Marques-Vidal, P., Bochud, M., Bastardot, F., Luscher, T., Ferrero, F., Gaspoz, J.M., Paccaud, F., Urwyler, A., von Kanel, R., Hock, C., Waeber, G., Preisig, M., and Vollenweider, P., *Levels and determinants of inflammatory biomarkers in a Swiss population-based sample (CoLaus study).* PLoS One, 2011. 6(6): p. e21002. <https://doi.org/10.1371/journal.pone.0021002>

19. Manichaikul, A., Mychaleckyj, J.C., Rich, S.S., Daly, K., Sale, M., and Chen, W.-M., *Robust relationship inference in genome-wide association studies.* Bioinformatics, 2010. 26(22): p. 2867-2873.

20. Delaneau, O., Marchini, J., and Zagury, J.-F., *A linear complexity phasing method for thousands of genomes.* Nature methods, 2012. 9(2): p. 179-181.

21. Delaneau, O., Zagury, J.-F., and Marchini, J., *Improved whole-chromosome phasing for disease and population genetic studies.* Nature methods, 2013. 10(1): p. 5-6.

22. Das, S., Forer, L., Schönherr, S., Sidore, C., Locke, A.E., Kwong, A., Vrieze, S.I., Chew, E.Y., Levy, S., and McGue, M., *Next-generation genotype imputation service and methods.* Nature genetics, 2016. 48(10): p. 1284-1287.

23. McCarthy, S., Das, S., Kretzschmar, W., Delaneau, O., Wood, A.R., Teumer, A., Kang, H.M., Fuchsberger, C., Danecek, P., Sharp, K., Luo, Y., Sidore, C., Kwong, A., Timpson, N., Koskinen, S., Vrieze, S., Scott, L.J., Zhang, H., Mahajan, A., Veldink, J., Peters, U., Pato, C., van Duijn, C.M., Gillies, C.E., Gandin, I., Mezzavilla, M., Gilly, A., Cocca, M., Traglia, M., Angius, A., Barrett, J.C., Boomsma, D., Branham, K., Breen, G., Brummett, C.M., Busonero, F., Campbell, H., Chan, A., Chen, S., Chew, E., Collins, F.S., Corbin, L.J., Smith, G.D., Dedoussis, G., Dorr, M., Farmaki, A.E., Ferrucci, L., Forer, L., Fraser, R.M., Gabriel, S., Levy, S., Groop, L., Harrison, T., Hattersley, A., Holmen, O.L., Hveem, K., Kretzler, M., Lee, J.C., McGue, M., Meitinger, T., Melzer, D., Min, J.L., Mohlke, K.L., Vincent, J.B., Nauck, M., Nickerson, D., Palotie, A., Pato, M., Pirastu, N., McInnis, M., Richards, J.B., Sala, C., Salomaa, V., Schlessinger, D., Schoenherr, S., Slagboom, P.E., Small, K., Spector, T., Stambolian, D., Tuke, M., Tuomilehto, J., Van den Berg, L.H., Van Rheenen, W., Volker, U., Wijmenga, C., Toniolo, D., Zeggini, E., Gasparini, P., Sampson, M.G., Wilson, J.F., Frayling, T., de Bakker, P.I., Swertz, M.A., McCarroll, S., Kooperberg, C., Dekker, A., Altshuler, D., Willer, C., Iacono, W., Ripatti, S., Soranzo, N., Walter, K., Swaroop, A., Cucca, F., Anderson, C.A., Myers, R.M., Boehnke, M., McCarthy, M.I., Durbin, R. and Haplotype Reference, C., *A reference panel of 64,976 haplotypes for genotype imputation.* Nat Genet, 2016. 48(10): p. 1279-83. <https://doi.org/10.1038/ng.3643>

24. Choi, S.W. and O'Reilly, P.F., *PRSice-2: Polygenic Risk Score software for biobank-scale data.* Gigascience, 2019. 8(7): p. giz082.

25. Wray, N.R., Ripke, S., Mattheisen, M., Trzaskowski, M., Byrne, E.M., Abdellaoui, A., Adams, M.J., Agerbo, E., Air, T.M., and Andlauer, T.M., *Genome-wide association analyses identify 44 risk variants and refine the genetic architecture of major depression.* Nature genetics, 2018. 50(5): p. 668-681.

26. Howard, D.M., Adams, M.J., Shirali, M., Clarke, T.-K., Marioni, R.E., Davies, G., Coleman, J.R., Alloza, C., Shen, X., and Barbu, M.C., *Genome-wide association study of depression phenotypes in UK Biobank identifies variants in excitatory synaptic pathways.* Nature communications, 2018. 9(1): p. 1470.

27. Zhao, S., Bao, Z., Zhao, X., Xu, M., Li, M.D., and Yang, Z., *Identification of diagnostic markers for major depressive disorder using machine learning methods.* Frontiers in Neuroscience, 2021. 15: p. 645998.
